# Incidence of Norovirus-Associated Diarrhea, Shanghai, China, 2012–2013

**DOI:** 10.3201/eid2302.161153

**Published:** 2017-02

**Authors:** Jianxing Yu, Chuchu Ye, Shengjie Lai, Weiping Zhu, Zike Zhang, Qibin Geng, Caoyi Xue, Weizhong Yang, Shuyu Wu, Aron J. Hall, Qiao Sun, Zhongjie Li

**Affiliations:** Division of Infectious Disease, Key Laboratory of Surveillance and Early-Warning on Infectious Disease, Chinese Center for Disease Control and Prevention, Beijing, China (J. Yu, S. Lai, Z. Zhang, Q. Geng, W. Yang, Z. Li);; Ministry of Health Key Laboratory of Systems Biology of Pathogens and Dr. Christophe Mérieux Laboratory, CAMS-Fondation Mérieux, Institute of Pathogen Biology, Academy of Medical Sciences of China and Peking Union Medical College, Beijing (J. Yu);; Research Base of Key Laboratory of Surveillance and Early-Warning on Infectious Disease, Pudong New Area Center for Disease Control and Prevention, Chinese Center for Disease Control and Prevention, Shanghai, China (C. Ye, W. Zhu, C. Xue, Q. Sun);; State Key Laboratory for Diagnosis and Treatment of Infectious Diseases, First Affiliated Hospital, Zhejiang University School of Medicine, Hangzhou, China (Z. Zhang);; State Key Laboratory of Virology, Wuhan University College of Life Sciences, Wuhan, China (Q. Geng);; Centers for Disease Control and Prevention, Beijing, China (S. Wu);; Centers for Disease Control and Prevention, Atlanta, Georgia, USA (A.J. Hall)

**Keywords:** norovirus, viruses, incidence rate, acute gastroenteritis, diarrhea, outpatients, sentinel surveillance, Pudong, Shanghai, China, enteric infections

## Abstract

We conducted sentinel-based surveillance for norovirus in the Pudong area of Shanghai, China, during 2012–2013, by analyzing 5,324 community surveys, 408,024 medical records, and 771 laboratory-confirmed norovirus infections among 3,877 diarrhea cases. Our analysis indicated an outpatient incidence of 1.5/100 person-years and a community incidence of 8.9/100 person-years for norovirus-associated diarrhea.

Norovirus is the most common cause of gastroenteritis (i.e., diarrhea or vomiting) (*1*). Diarrhea represents the second greatest burden of infectious disease in the world, and globally, ≈20% of diarrhea cases are associated with norovirus infection (*2*). China is one of the 15 highest-burden countries for diarrhea in the world (*3*). To guide the planning, implementation, and evaluation of disease control programs, nationwide sentinel-based surveillance for diarrhea across all age groups has been conducted in China since 2009, in which the prevalence of norovirus is monitored regularly (*4*). However, incidence rates of norovirus are not readily available from previous studies because of the lack of population denominators (*4*,*5*). To assess the population-based burden of norovirus disease based on this surveillance platform, we conducted community surveys and reviewed medical records for diarrhea in the Pudong New Area (a district of the city of Shanghai, China) during 2012–2013. We then estimated age-stratified rates for norovirus-associated diarrhea.

## The Study

We conducted surveillance for diarrhea (defined as >3 passages of watery, loose, bloody, or mucoid stools within a 24-hour period) at outpatient clinics (mainly enteric, pediatric, and internal medicine clinics) of 10 sentinel hospitals in Pudong during February 1, 2012–December 31, 2013. We determined the catchment population, market share of sentinel hospitals, and weighted proportion of persons with diarrhea in the community who sought medical care by conducting the age-stratified Hospital Utilization and Attitudes Survey among respondents residing in Pudong (Technical Appendix). In total, an estimated population of 1,722,580 was under surveillance during the study period.

**Figure 1:**
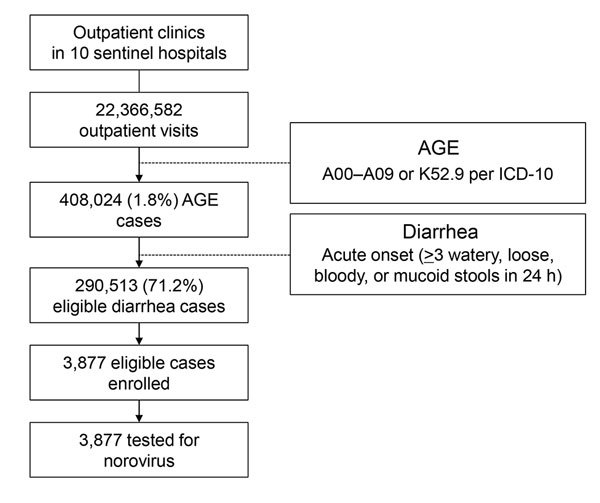
Registration, enrollment, and testing of diarrhea case patients in Pudong New Area, Shanghai, China, 2012–2013. (A pilot study was conducted during the first month of the year 2012. No case enrollment was conducted during that period.) AGE, acute gastroenteritis; ICD-10, International Classification of Diseases, 10th Revision.

We obtained the number of outpatient visits for diarrhea each year at 10 sentinel hospitals by reviewing and analyzing medical records. We extracted medical records of acute gastroenteritis (AGE), encoded as A00–A09 or K52.9 by the International Classification of Diseases, 10th Revision (ICD-10), from 10 sentinel hospitals’ hospital information systems (HIS) in 2012 and 2013. In total, 408,024 episodes of AGE were identified (189,645 in 2012 and 218,379 in 2013). To validate these AGE records with diarrhea cases likely meeting the study case definition, we ascertained numbers of diarrhea cases at 7 sentinel hospitals’ enteric clinics during February 2012–December 2013 (Technical Appendix). We identified 39,365 AGE episodes at these clinics and identified 28,030 eligible diarrhea case-patients during the same period. We used the ratio of 0.712 (28,030/39,365) as a contractive factor to narrow down the ICD-10–coded AGE episodes to diarrhea cases likely meeting the study case definition (Figure 1). After validation, we estimated that 290,513 (408,024 × 0.712) diarrhea case-patients visited the 10 sentinel hospitals in 2012 and 2013.

**Figure 2:**
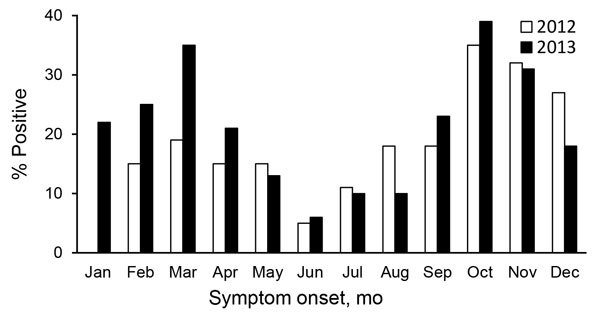
Proportion of norovirus detected among diarrhea case patients in outpatient settings, by month of symptom onset, Pudong New Area, Shanghai, China, 2012–2013.

To measure the proportion of diarrhea episodes associated with norovirus, physicians working at outpatient clinics of the 10 sentinel hospitals screened visiting patients for eligibility of enrollment; the first 1–3 eligible diarrhea case-patients for each week in each sentinel hospitals were recruited by using a convenience sampling method. Fecal specimens were collected for enrolled case-patients and tested for norovirus genogroups GI and GII in the local public health laboratory by using reverse transcription PCR (RT-PCR) assays as described previously (*4*). In total, we enrolled 3,877 diarrhea case-patients (2,235 in 2012 and 1,642 in 2013). We detected norovirus in 771 cases (19.9%). We observed no significant difference in norovirus detection between surveillance years (19% in 2012 vs. 21% in 2013). However, detection of norovirus was significantly different between age groups (22% in persons >5 years of age vs. 12% in children <5 years of age; p<0.001) (Table 1). The highest norovirus detection rates were in October (35%–38%) and the lowest in June (5%–6%), but a secondary peak in detection occurred in March (19%–34%) (Figure 2).

**Table 1:** Norovirus test results among diarrhea case-patients, by sex, surveillance year, and age group, Pudong New Area, Shanghai, China, 2012–2013

Variable	Positive, no. (%)	Negative, no. (%)	p value
Total	771 (100)	3,106 (100)	
Sex			0.278
M	426 (21)	1,646 (79)	
F	345 (19)	1,460 (81)	
Surveillance year			0.074
2012	422 (19)	1,813 (81)	
2013	349 (21)	1,293 (79)	
Age group			<0.001
0–11 mo	30 (10)	277 (90)	
12–23 mo	23 (16)	124 (84)	
24–59 mo	28 (13)	184 (87)	
5–24 y	69 (18)	306 (82)	
25–44 y	268 (21)	1,028 (79)	
45–64 y	263 (24)	828 (76)	
>65 y	90 (20)	359 (80)	

We calculated outpatient incidence rates for norovirus-associated diarrhea in Pudong as the number of outpatient visits associated with norovirus divided by the total population at risk, which was based on total episodes of AGE in sentinel hospitals, the contractive factor used to narrow down the total episodes of ICD-10–coded AGE to diarrhea cases likely meeting the study case definition, the percentage of diarrhea case-patients with norovirus, and the total study catchment population. We estimated community incidence rates by dividing the number of outpatient visits associated with norovirus by the product of population at risk and the proportion of persons with diarrhea in the community who sought medical care, based on results of the Hospital Utilization and Attitudes Survey. We calculated 95% CIs by using bootstrap methods with 1,000 samples for each rate.

We estimated the annual outpatient incidence rates of norovirus-associated diarrhea in Pudong to be 1.3/100 person-years (95% CI 1.2–1.4) in 2012 and 1.6/100 person-years (95% CI 1.4–1.8) in 2013, with an average rate of 1.5/100 person-years (95% CI 1.4–1.6) for the 2 years combined (Table 2). Children <2 years of age (7.4/100 person-years, 95% CI 5.4–9.3) and adults >65 years of age (2.6/100 person-years, 95% CI 2.0–3.2) exhibited increased rates of outpatient visits, compared with adults 25–44 years of age (1.0/100 person-years, 95% CI 0.9–1.1) (Table 2). The annual community incidence rates for norovirus-associated diarrhea were 8.1/100 person-years (95% CI 7.2–9.0) in 2012 and 9.8/100 person-years (95% CI 8.7–11.0) in 2013, with an average rate of 8.9/100 person-years (95% CI 8.2–9.7) for the 2 years combined.

**Table 2:** Estimated outpatient and community incidence of norovirus-associated diarrhea, by surveillance year, age group of patients, and season, Pudong New Area, Shanghai, China, 2012–2013

Variable	Outpatient incidence, no. cases/100 person-years (95% CI)	Community incidence, no. cases/100 person-years (95% CI)
Surveillance year		
2012	1.3 (1.2–1.4)	8.1 (7.2–9.0)
2013	1.6 (1.4–1.8)	9.8 (8.7–11.0)
2 y combined	1.5 (1.4–1.6)	8.9 (8.2–9.7)
Age group		
0–11 mo	8.4 (5.8–11.6)	20.3 (13.8–27.9)
12–23 mo	6.8 (4.2–9.6)	14.7 (9.2–20.8)
24–59 mo	1.4 (1.0–2.0)	2.5 (1.7–3.5)
5–24 y	1.1 (0.8–1.3)	8.8 (7.0–10.8)
25–44 y	1.0 (0.9–1.1)	13.0 (11.6–14.7)
45–64 y	1.7 (1.5–1.9)	3.2 (2.8–3.6)
>65 y	2.6 (2.0–3.2)	7.8 (6.1–9.5)
Season		
Spring, Mar–May	1.2 (1.0–1.4)	7.9 (6.7–9.0)
Summer, Jun–Aug	0.8 (0.7–1.0)	4.9 (3.9–6.0)
Autumn, Sep–Nov	2.3 (2.1–2.5)	13.4 (11.7–15.1)
Winter, Dec–Feb	1.7 (1.4–1.9)	10.5 (8.8–12.3)

## Conclusions

This 2-year study provides age-stratified incidence rates for norovirus infections among medically attended diarrhea patients in Pudong, Shanghai. We used a sentinel-based surveillance approach and combined multiple data sources to generate incidence, including 5,324 community surveys, 408,024 retrospectively collected electronic medical records, and 771 laboratory-confirmed norovirus infections identified among 3,877 diarrhea cases. Each year in Pudong, ≈1 in 11 persons became ill with norovirus-associated diarrhea in the community (corresponding to 526,000 cases) and ≈1 in 67 persons visited a healthcare provider for norovirus-associated diarrheal illness (corresponding to 88,000 ambulatory visits). These findings suggest that norovirus was a substantial burden on the community and healthcare system of Pudong.

In our study, outpatient rates of norovirus-associated diarrhea were consistent between the 2 consecutive surveillance years of 2012 and 2013. The average annual incidence for norovirus-associated outpatient visits was estimated to be 1.5/100 person-years, which was broadly consistent although slightly higher than that reported in studies conducted in other countries, such as the United States (0.4–1.2/100 person-years) (*6*), the United Kingdom (0.48–0.6/100 person-years) (*7*), Germany (0.29–1.07/100 person-years) (*8*), and the Netherlands (0.5–1.5/100 person-years) (*9*). The average prevalence of norovirus across all age groups in our study was 19.9%, similar to the global estimate of 20% from outpatient studies by Ahmed et al. (*2*). This prevalence was higher when compared with some other studies that were conducted in other countries (4.4%–16%) (*6*–*8*). We believe that high exposure to norovirus in our study population might have been a source of the elevated rates. The new norovirus GII.4 variant Sydney_2012 emerged during our study period and has caused several outbreaks in eastern China since 2012 (*10*,*11*). Although sequencing information was lacking in our study and we have only 2 years of surveillance data, given the elevated rates and substantial burden of norovirus in our study population, we should remain vigilant and continue to monitor norovirus, ideally with genotype information, in future studies.

Technical Appendix Description of Hospital Utilization and Attitudes Survey (HUAS). Methods for retrieval and validation of acute gastroenteritis records from hospital information systems of 10 sentinel hospitals in Pudong New Area, Shanghai, China, 2012–2013.
