# Supplementary material for: Incidence of Norovirus-Associated Diarrhea, Shanghai, China, 2012–2013
Source: Emerg Infect Dis. 2017 Feb;23(2):312–5. doi: 10.3201/eid2302.161153 (PMC5324797; doi:10.3201/eid2302.161153)
Supplement: Technical Appendix — Description of Hospital Utilization and Attitudes Survey (HUAS). Methods for retrieval and validation of acute gastroenteritis records from hospital information systems of 10 sentinel hospitals in Pudong New Area, Shanghai, China, 2012–2013. [file 16-1153-Techapp-s1.pdf]

Article DOI: http://dx.doi.org/10.3201/eid2302.161153

Incidence of Norovirus-Associated Diarrhea, Shanghai, China, 2012–2013

Technical Appendix

Hospital Utilization and Attitudes Survey (HUAS)

To define the catchment population for 10 hospitals that conducted diarrhea surveillance from February 1, 2012 through December 31, 2013 at Pudong New Area of Shanghai City, a cross-sectional survey (hospital utilization and attitudes survey [HUAS]) was conducted among 5,324 community residents of Pudong. Face-to-face interviews were conducted separately among 2 age strata, children aged <5 years and people aged ≥5 years. Diarrhea episodes (defined as ≥3 passages of watery, loose, bloody, or mucoid stools within a 24-hour period) and health care utilization practices (whether they consulted a doctor and where they consulted) in the previous 4 weeks before interview were assessed.

HUAS results are shown in Technical Appendix Table. The weighted monthly prevalence of diarrhea in the community of Pudong was 4.1% (95% CI 3.6–4.7), corresponding to an incidence rate of 0.54 episodes/person-year. Among respondents reporting one or more newly occurred episode of diarrhea in the previous month, 21.2% reported that they had consulted a doctor or sought medical care as a result of the diarrhea. In total, 1,722,580 persons were estimated to comprise the catchment of these 10 hospitals that conducted surveillance for diarrhea during 2012–2013 (Technical Appendix Table). The catchment population consisted of 29% of the registered population in Pudong, and was very close to the market share of these 10 hospitals (27% of total population in Pudong) (*1*), calculated as the outpatient/emergency department visits of 10 hospitals (10.1 million) divided by the total outpatient/emergency department visits of Pudong (37.5 million) in year 2012.

Retrieving and Validation of Acute Gastroenteritis (AGE) Records from the Hospital Information System (HIS) of 10 Sentinel Hospitals (SHs) in Pudong

Retrieving of AGE records

To help measure occurrence and characterize distribution of diarrhea cases by time, place, and population, data on outpatient visits were obtained from the Hospital Information System (HIS) of 10 sentinel hospitals (SHs) that conducted surveillance for diarrhea in Pudong during February 1, 2012 to December 31, 2013. Data were anonymized by dropping the name of patients before use so that the identity of any individual could not be uncovered. Acute gastroenteritis (AGE) records (encoded as A00–A09 or K52.9 by the *International Classification of Diseases, 10th Revision*, or had any of the terms “diarrhea, enteritis, gastroenteritis, ileitis, jejunitis or sigmoiditis” in diagnoses) were extracted and analyzed for study purposes.

During our study period of 2012–2013, 408,024 (2%) records of AGE were retrieved from 22,366,582 records of outpatient visits in HIS of 10 SHs in Pudong (Technical Appendix Table 2). 189,645 (46%) AGE visits were obtained in 2012, and 218,379 (54%) in 2013. Most (32%) of the AGE visits were among children aged <5 years (Technical Appendix Figure 1). The seasonal pattern for occurrence of AGE visits was different by age group, with peaks in winter months for children aged <5 years and peaks in summer for other age groups (Technical Appendix Figure 2).

Validation of AGE Records

To help validate retrospectively collected AGE records, eligible diarrhea case patients (defined as $\geq$3 passages of watery, loose, mucoid, or bloody stools within a 24 hour period) were also reported in selected outpatient clinics (enteric clinics) at 7 SHs during the study period, which were selected based on the willingness of the hospitals to participate. The ratio of reported diarrhea cases to ICD-coded AGE records from HIS in the same enteric clinics during 2012–2013 were used to scale down the total episodes of AGE from any cause to newly occurred diarrhea cases likely meeting our study case definition.

During our study period of 2012–2013, 28,030 cases of eligible diarrhea were reported at enteric clinics of the 7 SHs. In the same period, 39,365 ICD-coded records of AGE were retrieved from the HIS of the same enteric clinics. Thus, 71.2% (28030/39365) of ICD-coded AGE episodes were likely eligible diarrhea cases for inclusion in our surveillance study. This

ratio (0.712) was used as a contractive factor to scale down the total episodes of ICD-coded AGE among all 10 SHs included in the full surveillance study to the estimated number of newly occurred diarrhea cases likely meeting our case definition.

Reference

1. Xiaojun G, Jizeng L. Shanghai Pudong new area statistical yearbook. Beijing: China Statistics Press; 2012.

Technical Appendix Table 1. Healthcare utilization practices of diarrhea cases in the community of Pudong New Area, Shanghai, China, by age group*

Age group	% Persons reported one or more newly occurred episodes of diarrhea in previous 4 weeks	% Persons with diarrhea who sought medical care	Estimated no. of persons who sought medical care for diarrhea at the 10 hospitals (% of total within the age group)†
0–11 mo, n = 397	11	42	25,594 (65)
12–23 mo, n = 458	6	46	31,934 (62)
24–59 mo, n = 1,348	3	58	96,040 (63)
5–24 year, n = 671	4	12	362,969 (30)
25–44 year, n = 1,372	5	8	701,827 (29)
45–64 year, n = 818	3	45	385,733 (25)
≥65 year, n = 274	5	45	118,483 (23)
Total, N = 5,338	4‡	21	1,722,580 (29)

*Unpublished data.
†The proportions in the brackets were calculated as the number of respondents who answered that they would like to seek medical care for diarrhea at the 10 hospitals divided by the total number of respondents interviewed within that age group. When multiplied by the registered population number at the middle term of year 2012 in Pudong, it produces the estimated number of catchment population in the column.
‡Weighted monthly prevalence.

Technical Appendix Table 2. Outpatient visits with acute gastroenteritis (AGE)* diagnosed in 10 sentinel hospitals, Pudong New Area, Shanghai, China, 2012–2013

Age group	No. outpatient visits with AGE diagnoses	Total no. visits	AGE proportion of total, %
0–11 mo	59,182	802,997	7.4
12–23 mo	43,881	619,728	7.1
24–59 mo	28,980	1,482,024	2.0
5–24 y	58,999	3,007,685	2.0
25–44 y	95,614	5,083,752	1.9
45–64 y	77,137	6,249,896	1.2
≥65 y	43,078	4,973,896	0.9
Age unspecified	1,153	146,604	0.8
Total	408,024	22,366,582	1.8

*Acute gastroenteritis (AGE) = hospital outpatient medical records encoded as A00–A09 or K52.9 by the *International Classification of Diseases, 10th Revision*, or had characters “diarrhea, enteritis, gastroenteritis, ileitis, jejunitis or sigmoiditis” in diagnoses.

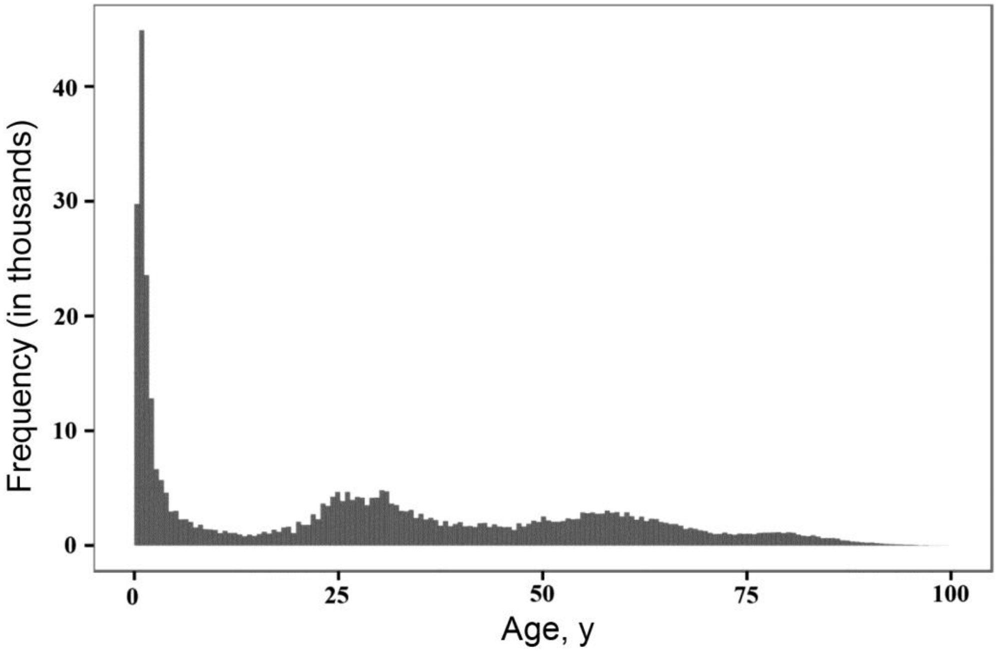

Technical Appendix Figure 1. Age distribution of outpatient visits with acute gastroenteritis, Pudong New Area, Shanghai, China, 2012–2013

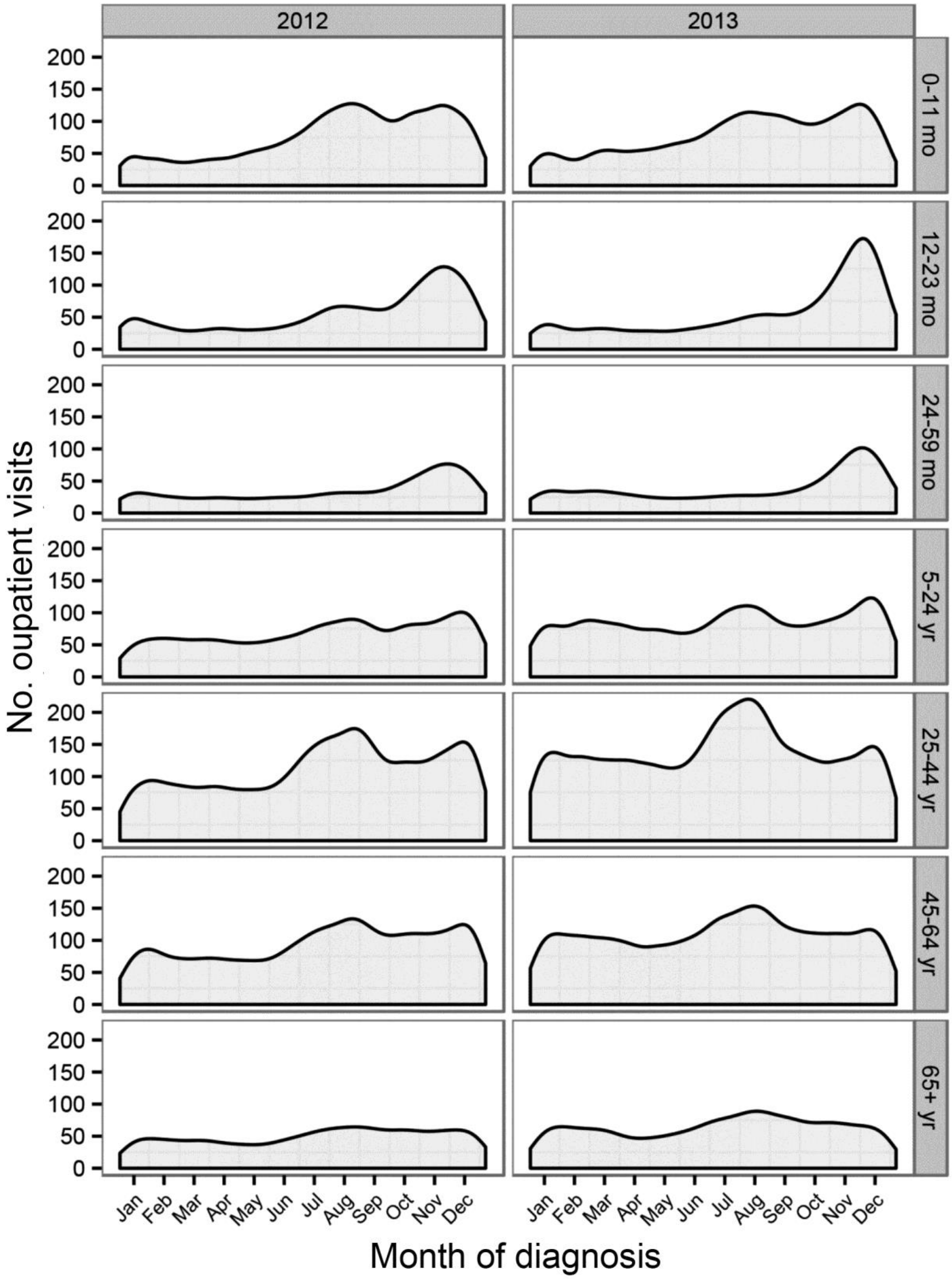

Technical Appendix Figure 2. Number of outpatient visits with acute gastroenteritis, by date of diagnosis and age group, Pudong New Area, Shanghai, China, 2012–2013
